# Label-free quantification of protein binding to lipid vesicles using transparent waveguide evanescent-field scattering microscopy with liquid control

**DOI:** 10.1364/BOE.490051

**Published:** 2023-07-10

**Authors:** Mokhtar Mapar, Mattias Sjöberg, Vladimir P. Zhdanov, Björn Agnarsson, Fredrik Höök

**Affiliations:** 1 Division of Biological Physics, Department of Physics, Chalmers University of Technology, SE-41296 Göteborg, Sweden; 2 Nanolyze AB, BioVentureHub, Pepparedsleden 1, SE-43183 Göteborg, Sweden; 3 Boreskov Institute of Catalysis, Russian Academy of Sciences, Novosibirsk 630090, Russia

## Abstract

Recent innovations in microscopy techniques are paving the way for label-free studies of single nanoscopic biological entities such as viruses, lipid-nanoparticle drug carriers, and even proteins. One such technique is waveguide evanescent-field microscopy, which offers a relatively simple, yet sensitive, way of achieving label-free light scattering-based imaging of nanoparticles on surfaces. Herein, we extend the application of this technique by incorporating microfluidic liquid control and adapting the design for use with inverted microscopes by fabricating a waveguide on a transparent substrate. We furthermore formulate analytical models describing scattering and fluorescence intensities from single spherical and shell-like objects interacting with evanescent fields. The models are then applied to analyze scattering and fluorescence intensities from adsorbed polystyrene beads and to temporally resolve cholera-toxin B (CTB) binding to individual surface-immobilized glycosphingolipid G_M1_ containing vesicles. We also propose a self-consistent means to quantify the thickness of the CTB layer, revealing that protein-binding to individual vesicles can be characterized with sub-nm precision in a time-resolved manner.

## 1. Introduction

The rapid progress witnessed in many branches of life science goes hand-in-hand with continuous development and improvements of bioanalytical instrumentation. The emergence of surface-sensitive optical microscopy techniques that enable the study of dynamic biological processes down to single biomolecules has, for the past decade, been fueling a continual progression within basic science, medical diagnostics, and drug discovery [1–4]. A key feature of such techniques is their capability to study single biological entities, revealing detailed information on population heterogeneities – information that is typically hidden in ensemble-average based methods. Most work on time-resolved single-particle imaging relies on some form of labeling strategy, e.g. by fluorophores, quantum dots, or metallic nanoparticles; however, label-free imaging and quantitative analysis of nanoscale biological assemblies such as viruses and even individual proteins have recently been accomplished using evanescent-light microscopy [5,6] and/or interferometric microscopy schemes [7–9], which alone, or in combination with fluorescence readout opens up entirely new opportunities in biological imaging.

Evanescent-field illumination is most commonly realized using either prism- or objective-based total internal reflection (TIR) and is mainly applied to visualize fluorescently labelled objects in close proximity to a solid-liquid interface [10]. The high surface confinement of the illumination profile achieved in this way significantly improves surface sensitivity and imaging contrast compared to standard epi-illumination and confocal-based microscopy, allowing for binding kinetics of single fluorescent molecules or nanoparticles to be studied, even in the presence of appreciable amounts of these species in the bulk [11–13].

Another approach for performing evanescent field microscopy is to use planar optical waveguides, where the cladding around a waveguide core has been partly replaced with the sample to be investigated [14–22]. With waveguide illumination, the paths of the illumination and detection signals are independent and essentially perpendicular to each other. Consequently, there are no special requirements on the objectives used for signal collection, as is the case for in-lens TIR where specially designed high magnification, high numerical aperture objectives, and specific optical-path control units must be employed. Furthermore, compared to both prism- and objective-based TIR, waveguides can produce large and uniform areas of illumination, allowing the use of low-magnification objectives and thus observations with a larger field-of-view [20,21].

As with prism- or objective based TIR, waveguide microscopy is most commonly applied for studying fluorescently labeled objects [14–17,19,22], and has recently been demonstrated to offer large field-of-view fluorescence super-resolution imaging by taking advantage of multimode interference patterns within the waveguide structure [21,23]. However, due to an inherent low stray-light scattering (background scattering), waveguides have also been applied for label-free light scattering-based imaging of biological nanoparticles, thus offering clear advantages in cases where labelling of biological particles is challenging or unfavorable [18,24–26].

**Fig. 1.**
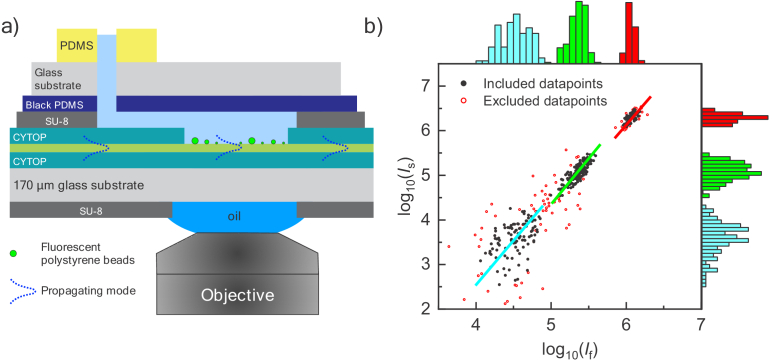
a) A schematic (not to scale) of the waveguide chip with the incorporated microfluidic channels. b) Logarithmic representation of scattering versus fluorescence intensity of three different populations of surface adsorbed fluorescent polystyrene beads (radius of: 26 nm colored in cyan, 50 nm colored in green and 94 nm colored in red) obtained with waveguide illumination. The red hollow circles represent excluded data-points that are more than three standard deviations away from the center of a fitted bivariate lognormal distribution of each cluster. The straight cyan, green and red lines are the principle-components of the selected data points (black solid circles) of the three observed clusters, with slopes of 1.98, 1.87 and 1.70, respectively. The corresponding intensity distributions are projected on its respective axis.

In the present work, we report a waveguide structure (Fig. 1(a)), fabricated on a transparent, sub-200 µm glass slide to make it compatible for use with inverted microscopes and high numerical aperture oil-immersion objectives. The structure furthermore incorporates a microfluidic channel that facilitates easy and effective liquid exchange. To facilitate the interpretation of the information provided by the waveguide, we present and extend a set of models describing fluorescence and scattering intensity from sub-micrometer particles interacting with an evanescent field. The waveguide’s performance and applicability of the models are tested by measuring and theoretically scrutinizing the scattering and fluorescence intensities from individual 25-100 nm in radius fluorescent polystyrene beads adsorbed to the surface of the waveguide ship. As a more complex example of the interplay of measurements and theory, we subsequently apply our approach to estimate the thickness of a cholera-toxin B (CTB) subunit layer bound to glycosphingolipid G_M1_ modified lipid vesicles in a label-free manner. This was achieved by temporally monitoring changes in scattering intensity of individual vesicles upon CTB binding and relating the change in intensities to an increase in CTB-lipid shell thickness. Taken together, these results demonstrate how microfluidics combined with label-free light-scattering imaging facilitates time-resolved quantification of protein binding to individual biological nanoparticles. We also scrutinize the accuracy of the quantification as a function of vesicle radius and protein film thickness.

## 2. Materials and methods

### 2.1 Transparent waveguide chip

Transparent waveguide chips with integrated fluidic channels were produced in a similar fashion as previously reported [27], but this time on a transparent glass substrate. This extends the waveguide compatibility to inverted microscopes and oil-immersion objectives and facilitates easy access to the sample area (Fig. 1(a)). To further minimize stray light scattering and to attenuate stray light in the transparent substrate, a black-dyed layer was patterned on the back of the substrate using photolithography methods. For a detailed description of the fabrication process of the waveguide chips, see Sec. 1 in Supplement 1.

### 2.2 Microscopic setup and coupling of light to the waveguide

A polarization-maintaining single-mode fiber with 3.5 µm in diameter core layer, PM460-HP (Thorlabs Inc.) was cut, stripped, and aligned to deliver TE-polarized 488 nm laser light (Cobolt, 06-01 Series) to the waveguide by butt-coupling. The fiber was adjusted relative to the waveguide facet using a 3-axis translator mounted on the microscope stage [16]. The experiments were carried out using a Nikon Eclipse Ti microscope equipped with a CFI Plan Fluor 100XS oil objective with an adjustable iris (see Sec. 4 in Supplement 1 for additional measurements on polystyrene beads as a function of iris opening). Scattered and fluorescent light was collected simultaneously using an image-splitter [28] where it was separated using a dichroic mirror with a cut-off at 510 nm and then filtered using a 488/10 bandpass filter (scattered light) and 650/ (for bead measurements) or 535/50 (for FITC-CTB measurements). Images were acquired using either an Andor Neo or a Hamamatsu ORCA-Flash4.0 sCMOS camera.

### 2.3 G_M1_ conjugated vesicles, PLL-g-PEG and fluorescent beads

1-Palmitoyl-2-Oleoyl-sn-Glycero-3-Phosphocholine (POPC), a phospholipid, and 1,2-distearoyl-sn-glycero-3-phosphoethanolamine-N-[biotinyl(polyethylene glycol)-2000] (DSPE-PEG(2000)-biotin) were purchased from Avanti Polar Lipids (USA). Monosialoganglioside (G_M1_) from bovine brain and FITC conjugated Cholera-Toxin subunit B (CTB) were purchased from Sigma-Aldrich (Germany). Fluoro-Max red aqueous fluorescent polystyrene beads (25, 50 and 100 nm in nominal radius with an absorption maximum at 542 nm and emission maxima at 612 nm) were purchased from ThermoFisher Scientific (USA). The size of the polystyrene beads (Fig. S3 in Sec. 2 in Supplement 1) was evaluated using Nanoparticle Tracking Analysis (NTA; Malvern, UK) and was found to be normally distributed around 52, 100 and 188 nm, respectively. The polymers poly(L-lysine)-g-poly(ethylene glycol) [PLL(20)-g[3.5]-PEG(5)] (PLL-g-PEG for short), PLL(20)-g[3.5]-PEG(2)/PEGbiotin(3.4)50%, (PLL-g-PEG-biotin for short) and poly(L-lysine) (PLL) were purchased from SuSoS AG, Switzerland.

### 2.4 Vesicle preparation

POPC, G_M1_ and DSPE-PEG(2000)-biotin lipids were mixed in a desired molar ratio (95:4:1) to control the vesicle composition. The lipids were dissolved in a 50:50 mixture of chloroform and methanol, pipetted into a round bottom flask, dried under a gentle N_2_ stream and then placed in vacuum for 2 hours to obtain a lipid film. The dried film was then hydrated in a tris(hydroxymethyl)aminomethane (TRIS) buffer (20 mM TRIS, 100 mM NaCl, pH 7.5). Lipid vesicles were prepared by extruding the resulting lipid suspension through a 30 nm polycarbonate filter (Whatman, UK) 11 times. The expected size distribution of the extruded vesicles was confirmed using NTA (data not shown).

### 2.5 PLL-g-PEG polymer surfaces

Surfaces with self-assembled monolayers consisting of PLL-g-PEG polymers are commonly employed in bioanalytical sensor applications [29]. The polymer is frequently used to suppress unspecific binding, e.g. of liposomes and proteins, but can also be functionalized to incorporate target molecules of interest, such as biotin. To prepare surfaces with a desired surface coverage, suitable for single particle microscopy, mixtures of functional (PLL or PLL-g-PEG-biotin) and suppressing (PLL-g-PEG) polymers were prepared in known ratios at total concentration of 10 µg/ml and exposed to the silica core layer of the waveguide chip for 30 minutes before being thoroughly rinsed with deionized Milli-Q water.

### 2.6 Transparent waveguide chip

The waveguide chip designed in this work consists of a three-layered symmetric sandwich structure fabricated on a flat and optically transparent cover-glass substrate (see Fig. 1(a) and Sec. 1 in Supplement 1 for details). The upper and lower cladding layers of the waveguide are made from a fluorinated polymer (CYTOP) with a refractive index (*n* = 1.34) closely matching that of water (*n* = 1.33). The main benefit of this configuration is that it minimizes background light scattering and facilitates simple and easy light in-coupling [25–27,30]. The waveguide is furthermore designed to support only the fundamental optical mode, which ensures well-defined and controlled light confinement and propagation. An optical fiber is used to butt-couple a single mode, linearly polarized light into the chip, which results in evanescently decaying light at the boundary between the core and the cladding layers. The evanescent light is characterized by its penetration depth, defined as the distance where the square of the electric-field amplitude in the cladding layer has dropped to 1/e of its value at the core/cladding interface. To allow the sample under investigation to interact with the evanescent illumination, an opening of a desired size and shape is formed in the upper cladding layer which is thereby filled with the aqueous solution containing the specimen to be detected. The refractive index matching of cladding material with solution ensures minimal scattering within the measurement opening. An additional feature that distinguishes the current waveguide chip from other designs is that the core-layer is made from silica, which makes surface functionalization directly compatible with standard glass-surface-chemistries. Objects within the penetration depth will interact with the evanescent light via absorption and/or scattering, while objects outside the penetration depth will remain nearly unaffected. This configuration allows for specific monitoring of events occurring at, or close to, the interface between the glass core and the solution, thus enhancing signal-to-background contrast, and is therefore particularly effective when monitoring dynamic surface-binding events in complex solutions.

There is a mismatch between the fundamental modes of the 3.5 micron single-mode optical fiber and the 480 nm thick core layer of the planar waveguide, which may lead to stray light going into the CYTOP cladding and/or the cover-glass substrate and thus contributes to increased background. This problem is however mitigated by applying a thin layer of either black PDMS or black SU-8 photoresist on the waveguide as described in Sec. 1 in Supplement 1. Hence, due to the low stray-light (background) and since the optical paths of the excitation/incident and emitted/scattered light are decoupled, measurements can be performed by monitoring either the scattered evanescent light directly (*scattering mode*) or the emitted fluorescence light (*fluorescence mode)* provided appropriate optical filters are used.

Image processing was performed in the same manner as outlined in the Supplement 1 of Ref. [27], with local background subtracted from each particle at each timepoint (see Sec. 4 in Supplement 1 for distribution of mean local fluorescence and scattering background intensities).

## 3. Theory

In general, biological nanoparticles are complex in terms of size, shape, and molecular composition. In some cases, however, they can be viewed as either spheres or spherical shells of a particular refractive index between 1.4 and 1.6 [31,32]. Below, we present the analytical expressions describing fluorescence and scattering intensities of such structures interacting with an evanescent electromagnetic wave, propagating in a single-mode regime in the solution, adjacent to the waveguide core layer. In this wave, the electric field can be represented as [25,33]

(1) $$E=E_{0}\text{exp}[i\beta x-z/(2\delta )],\;$$

where $E_{0}$ is the amplitude at the interface, β is the wave propagation constant, δ is the penetration depth, and *x* and *z* are the coordinates along the propagation direction and perpendicular to the waveguide-solution interface, respectively.

The fluorescence emission from a spherical particle of radius $r_{0}$ , containing fluorescent dye of concentration *c*, uniformly distributed over its volume can be expressed as

(2) $$I_{\text{f}}=I_{\text{f}0}\eta_{\text{f},\text{ev}}\;,$$

where If0=AcI0(4π3)r03 is the emission calculated in the limit r0δ→0 $(I_{0}\propto E_{0}^{2}$ is the intensity of the incident light at z=0 , *A* a constant containing the absorption cross-section and fluorescence efficiency (fluorescence quantum yield) of the dye and $I_{0}$ is the intensity of the light interacting with the particle at the interface) and $\eta_{\text{f},\text{ev}}$ is the correction factor determined by the field extinction. The latter factor is given by Eqs. (10)–(12) in Ref. [34] and can be represented in a more compact form (compared to Ref. [34]) as

(3) $$\eta_{\text{f},\text{ev}}=3{\left(\frac{\delta }{r_{0}}\right)}^{3}\text{exp}\left(-\frac{r_{0}}{\delta }\right)\left[\frac{r_{0}}{\delta }\cosh\left(\frac{r_{0}}{\delta }\right)-\sinh\left(\frac{r_{0}}{\delta }\right)\right].$$

In contrast to fluorescence, the measured scattering intensity depends on not only the extinction of the evanescent field but also its phase. For a spherical particle, it can be calculated either numerically or analytically by extending the conventional Mie treatment [35]. Both these approaches are, however, cumbersome [36–38]. Explicit analytical expressions, which are sufficiently accurate, can be obtained by employing the Rayleigh-Gans-Debye (RGD) approximation [35] and extending it to account for the inherent field extinction [25]. The RGD approximation is applicable provided the relative (with respect to surrounding media) complex refractive index *m* is close to unity and the particle size is smaller than λ/|m−1 |, where λ is the wavelength [35]. The optical properties of biological matter are favorable from this perspective. Following this line, the scattering intensity of a spherical particle can be represented as

(4) $$I_{\text{s}}=\;I_{\text{s}0}\eta_{\text{s}}=\;B\alpha^{2}I_{0}\sin^{2}\left(\theta \right)\eta_{\text{s}},$$

where $I_{\text{s}0\;}=B\alpha^{2}I_{0}\sin^{2}(\theta )$ is the Rayleigh-scattering intensity, calculated neglecting the phase and extinction, *B* is a proportionality constant, α is the particle polarizability, θ is the angle between the propagation direction of the scattered light and the polarization vector of the incident light, and $\eta_{\text{s}}$ is the correction factor accounting for the phase shift and field extension. The latter factor can be obtained by numerical integration [12]. For applications, a more useful rather accurate approach is to factorize it as

(5) $$\eta_{\text{s}}=\eta_{\text{s},\text{ev}}\eta_{\text{RGD}}\;,$$

where $\eta_{\text{s},\text{ev}}$ and $\eta_{\text{RDG}}$ are the factors taking, respectively, the extinction and phase of the evanescent field into account. This approximation is reasonable provided each factor is not too small. The correction factor for the extinction can be calculated by analogy with Eq. (3) and represented as

(6) $$\eta_{\text{s},\text{ev}}=9{\left(\frac{2\delta }{r_{0}}\right)}^{6}\text{exp}\left(-\frac{r_{0}}{\delta }\right){\left[\frac{r_{0}}{2\delta }\cosh\left(\frac{r_{0}}{2\delta }\right)-\text{sinh}\left(\frac{r_{0}}{2\delta }\right)\right]}^{2},$$

whereas the latter factor is given by [35]

(7) $$\eta_{\text{RGD}}={\left|\frac{3}{{\left(ur_{0}\right)}^{3}}[\sin(ur_{0})-ur_{0}\cos(ur_{0})]\right|}^{2},$$

where u=2ksin(ϑ) (where $k=\beta n_{\text{m}}$ is the absolute value of the wave vector in a medium with refractive index $n_{\text{m}}$ and ϑ is the angle between the incident and scattered light).

In the case of vesicles or protein attachment to vesicles, the thickness of the lipid or lipid-protein layer is appreciably smaller than the light wave and extinction lengths, and accordingly the phase- and extinction-related corrections can be calculated assuming the thickness to be negligible. In particular, the fluorescence emission from a spherically shaped vesicles of radius $r_{0}$ , containing uniformly distributed fluorescent dye of surface concentration *N*, can be expressed as [39]

(8) $$I_{\text{f}}=\;I_{\text{f}0}\eta_{\text{f},\text{ev}}\;\;\text{with}\;\eta_{\text{f},\text{ev}}=\left[1-\text{exp}\left(-\frac{2r_{0}}{\delta }\right)\right]\delta /(2r_{0}),$$

where $I_{\text{f}0}\;=\;4\pi r_{0}^{2}ANI_{0}$ is the emission at $r_{0}/\delta \;\to \;0$ . In turn, the scattering is described by Eqs. (4) and (5) with

(9) $$\eta_{\text{s},\text{ev}}={\left[1-\text{exp}\left(-\frac{r_{0}}{\delta }\right)\right]}^{2}\delta^{2}/r_{0}^{2},$$

(10) $$\eta_{\text{RDG}}={\left[\frac{\sin(ur_{0})}{ur_{0}}\right]}^{2},$$

where *u* is defined as in Eq. (7). Equation (9) can be derived by analogy with Eq. (8) [25], whereas Eq. (10) can be found e.g. in Ref. [35].

If needed, the correction factors for the fluorescence and scattering emission from shell-like structures can be calculated taking the finite shell thickness into account. These factors depend on the shell external and internal (core) radii, $r_{\text{s}}$ and $r_{\text{c}}$ , and can be expressed via those or the corresponding amplitudes for spherical structures with these radii (see e.g. the RDG approach in Ref. [35]). For fluorescence, we have [cf. Equation (3)]

(11) $$\eta_{\text{f},\text{ev}}(r_{\text{s}},r_{\text{c}})=\frac{{r_{\text{s}}}^{3}}{{r_{\text{s}}}^{3}-{r_{\text{c}}}^{3}}\left[\eta_{\text{f},\text{ev}}(r_{\text{s}})-\frac{{r_{\text{c}}}^{3}\eta_{\text{f},\text{ev}}(r_{\text{c}})}{{r_{\text{s}}}^{3}}\text{exp}\left(-\frac{r_{\text{s}}-r_{\text{c}}}{\delta }\right)\right],$$

where $\eta_{\text{f},\text{ev}}(r_{\text{s}})$ is the extinction correction factor for a solid sphere given by Eq. (3). For scattering, the expressions are more cumbersome and can be represented as

(12) $$\eta_{\text{RDG}}(r_{\text{s}},r_{\text{c}})=\begin{array}{c} {\left|\frac{3}{{\left(ur_{\text{s}}\right)}^{3}-{\left(ur_{\text{c}}\right)}^{3}}[\left(\sin(ur_{\text{s}})-ur_{\text{s}}\cos(ur_{\text{s}}))-(\sin(ur_{\text{c}})-ur_{\text{c}}\cos(ur_{\text{c}})\right)]\right|}^{2}, \end{array}$$

(13) $$\begin{array}{c} \eta_{\text{s},\text{ev}}(r_{\text{s}},r_{\text{c}})=\\ 9{\left(\frac{{\left(2\delta \right)}^{3}}{{r_{\text{s}}}^{3}-{r_{\text{c}}}^{3}}\right)}^{2}\text{exp}\left(-\frac{r_{\text{s}}}{\delta }\right){\left[\frac{r_{\text{s}}}{2\delta }\cosh\left(\frac{r_{\text{s}}}{2\delta }\right)-\sinh\left(\frac{r_{\text{s}}}{2\delta }\right)-\left(\frac{r_{\text{c}}}{2\delta }\cosh\left(\frac{r_{\text{c}}}{2\delta }\right)-\sinh\left(\frac{r_{\text{c}}}{2\delta }\right)\right)\right]}^{2}. \end{array}$$

The equations presented above describe spherically symmetric structures. For less symmetric structures, it can be applied as well. Sometimes, it can be done analytically (e.g., in the case of $\eta_{\text{f}}$ or $\eta_{\text{s},\text{ev}}$ for pyramidally or conically shaped nanoparticles contacting the interface by their base). As a rule, however, it should be done numerically. For example, $\eta_{\text{f}}$ for nanoparticles with cylindrical symmetry, including thin nanotubes, nanorods (e.g., elongated viruses such as rhabdovirus), and $\eta_{\text{f}}$ of finite thickness (e.g., lipid tubules) can be found in Ref. [40] (the corresponding analysis was performed in the SPR/LSPR context).

## 4. Results

### 4.1 Fluorescent beads of different sizes

To evaluate the derived models for fluorescence and scattering intensities from spherical particles interacting with an evanescent field, we measured the scattering and fluorescence intensities for fluorescent polystyrene beads of three different radii (26, 50 and 94 nm) electrostatically adsorbed from aqueous suspensions to a PLL-g-PEG:PLL-functionalized waveguide-surface (see Materials and Methods). The PLL-g-PEG-part of the layer suppresses adsorption of beads while the positively charged PLL-part promotes electrostatic adsorption. Thus, by tuning the ratio of PLL to PLL-g-PEG, the amount of surface adsorbed beads could be easily controlled. For detailed characterization (size distribution and fluorescence intensity) of the polystyrene beads see Secs. 2-4 in Supplement 1.

The scattering and fluorescence intensities from over 400 beads were recorded using 40 ms and 2000 ms camera exposure, respectively. After accounting for variations in fluorescent dye concentrations between the three different bead sizes (see Sec. 3 in Supplement 1), the scattering and corresponding fluorescence intensities of the individual beads were plotted on a logarithmic scale to reveal subgroups of particles corresponding to the three bead sizes found on the surface (Fig. 1(b)). Data-points more than three standard deviations from the center of a fitted bivariate lognormal distribution were excluded from the following analysis (disregarded data is indicated with red hollow circles in Fig. 1(b)). Using principal component analysis (PCA), the datapoints could be fitted with a line that directly reveals the power-law relationship between the scattering and fluorescence intensities (see Fig. S6a in Supplement 1). The smallest beads (26 nm and 50 nm in radius) are only marginally affected by extinction [Eq. (6)] and phase shift [Eq. (7)] (see Fig. S6b in Supplement 1) and are thus expected to behave like point (Rayleigh) scatterers. This results in the PCA-lines having slopes close to two (2.0 and 1.9 for the 26 and 50 nm in radius beads, respectively), corresponding to the fluorescence intensity scaling with the bead volume and the scattering with the volume squared. The larger beads (94 nm in radius), however, are expected to be more affected by both phase and extinction, resulting in a PCA-line displaying a slope less than two. In our case the slope for the 94 nm beads is measured to 1.7, which is somewhat larger than the ≃1.5 expected based on theory (see Sec. 5 in Supplement 1 for a more detailed discussion).

Since the effects of extinction on fluorescence and scattering intensities [Eqs. (3) and (6), respectively] are very similar (see inset Fig. S6b in Supplement 1,), their relative effect in a logarithmic representation is partially cancelled leaving phase related effects as the main contribution to the deviation from a slope of two.

**Fig. 2.**
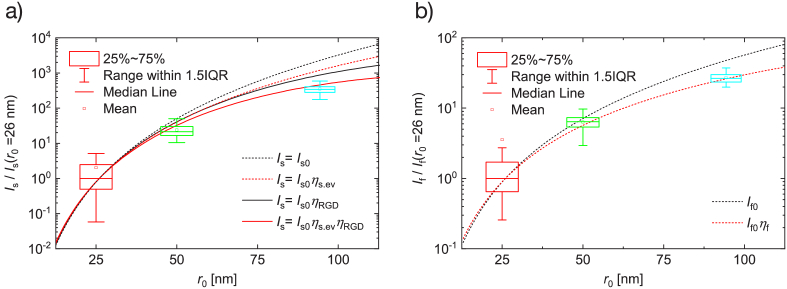
Scattering a) and fluorescence b) intensities from surface adsorbed fluorescent polystyrene beads as a function of their measured radii (26 nm, 50 nm and 94 nm). The intensities have been normalized to the median intensities for beads with $r_{0}=26$ nm [ $I_{\text{s}}(26\;\text{nm})$ and $I_{\text{f}}(26\;\text{nm})$ , respectively]. The data points show the results of measurements (the fluorescence values have been adjusted to account for difference in dye concentration between the differently sized beads, as described in Sec. 3 in Supplement 1) while the lines represent different adaptions of the proposed analytical models described in the text. In a), the models include $I_{\text{s}0}$ (black dashed line), $I_{\text{s}0}\eta_{\text{ev.s}}$ (red dashed line), $I_{\text{s}0}\eta_{\text{RGD}}$ (black solid line), and $I_{\text{s}0}\eta_{\text{ev.s}}\eta_{\text{RGD}}$ (red solid line). In b) two models are exhibited, one including the extinction correction factor, $I_{\text{f}0}\eta_{\text{f}}$ (red dotted line) and another neglecting it (black dotted line). The calculations were performed using δ=100 nm, k=0.018 nm^−1^ with integration performed over an angle from $\vartheta =41^{\circ }$ to $\vartheta =139^{\circ }$ corresponding to an objective with NA of 1.

To better understand the different contributions of extinction and phase shifts on the attained data, the measured scattering and fluorescence intensities were compared to different adaptations of the analytical models [Eqs. (2–7)]. When both correction factors are neglected ( $I_{\text{s}}=I_{\text{so}}$ and $I_{\text{f}}=I_{\text{fo}}$ ) the models tend to overestimate the scattering and fluorescence intensities (black dotted lines in Fig. 2(a) and 2(b)), while a better correspondence is obtained when including the full correction factors (Eqs. (6) and (7) in the case of scattering and Eq. (3) in the case of the fluorescence; red solid line in Fig. 2(a) and red dotted line in Fig. 2(b)). It is also evident how the effect of the correction factors increases with increasing bead size, with contribution of the phase-shift term dominating for the larger particles (see Sec. 5 in Supplement 1 for a detailed discussion on the contribution of each correction factor to the data).

The analysis presented indicates that the experimental data obtained for polystyrene nanobeads are in good agreement with the theory, provided all the corrections are taken into account. In the case of biological nanoparticles, the agreement is expected to be similar or even better because the refractive index of such particles better complies with the approximations used within the RGD theory.

### 4.2 Cholera-toxin B binding to glycosphingolipid G_M1_ conjugated vesicles

To assess the core-shell model [Eqs. (8–13)] and the sensitivity of the waveguide chip to detect and temporally resolve dynamic processes that conventionally requires fluorescence labelling, we studied protein binding to individual surface-bound shell-like nanoparticles. More specifically, we monitored specific binding of Cholera-toxin B (CTB) to lipid vesicles containing G_M1_ receptors. CTB is a 11 kDa subunit of the cholera toxin complex that assembles into pentamer-like structure with an approximate diameter of 2.4 nm and a length of 3 nm [41], which is known to form multivalent bonds to G_M1_ glycosphingolipids [42,43] frequently found on the lipid membranes of various cell types [44].

POPC vesicles containing 4 mol% G_M1_ and 1mol% biotin with a modal radius of ∼ 45 nm were bound to a PLL-g-PEG:PLL-g-PEG-biotin functionalized waveguide surface, using NeutrAvidin as inter-linkers. After subsequent rinsing, both scattering ( $I_{\text{s}}$ ) and fluorescence ( $I_{\text{f}}$ ) intensities of individual vesicles were simultaneously recorded as a function of time ( t ) at 400 ms exposure as they were exposed to a TE buffer containing 100 nM fluorescently labelled CTB. The data were then analyzed and used to evaluate the thickness of the bound CTB layer for each vesicle. The inert nature of the PLLgPEG layer suppresses non-specific adsorption of CTB to the surface while the POPC and DSPE-PEG(2000)-biotin lipids of the vesicles are recognized for their minimal non-specific binding characteristics [45].

**Fig. 3.**
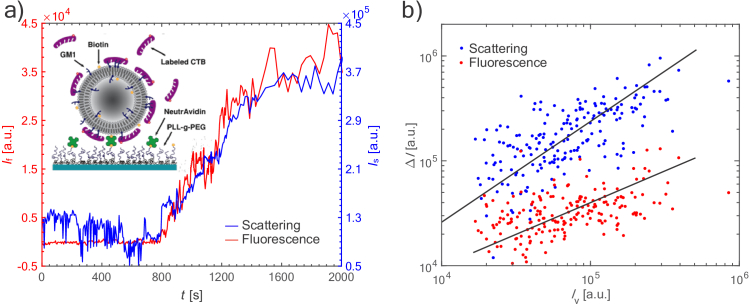
a) Representative example of CTB binding to a single POPC vesicle containing 4 mol% G_M1_ manifested simultaneously in increments of the fluorescence (red) and scattering (blue) intensities, $\Delta I_{\text{f}\;}$ and $\Delta I_{\text{s}\;}$ . The kinetics are shown for t≤2000 s. Typically, the measurements were performed at t≤3000 s (the data at t>3000 s do not add any new information). Vertical axes have been adjusted to match the respective data. The inset shows a schematic representation of the system. b) $\Delta I_{\text{s}\;}$ (blue dots) and $\Delta I_{\text{f}\;}$ (red dots) for individual vesicles after CTB binding as a function of the scattering intensity of the vesicle prior to CTB binding ( $I_{\text{s},\text{v}}$ ), in a logarithmic representation. The lines represent the principle-component of the corresponding data with slopes of 1.0 and 0.6 respectively.

Before introducing CTB to the system at 5 µl/min, the vesicles could only be monitored in scattering mode with many of them showing rapid intensity fluctuation (see scattering signal prior to onset of binding in Fig. 3(a) for t≤800 s), which we associate with vesicle dynamics and movements (wiggling and/or wobbling) [46]. After subsequent introduction of CTB (blue and red curves in Fig. 3(a), t=800 s) these rapid fluctuations were, for most vesicles, mitigated and replaced by a more gradual increase in both scattering and fluorescence intensities, which we associate with CTB binding to the vesicles. Both scattering and fluorescence intensities display similar binding kinetics and signal-to-noise levels until plateauing at $I_{\text{s},\;\;v+\text{CTB}}$ and $I_{\text{f},\text{CTB}}$ , respectively, indicating the end of CTB binding and a close-to-full CTB coverage (blue and red curves in Fig. 3(a), t>1600 s; see also Sec. 6 in Supplement 1 for additional examples of CTB binding to single vesicles). Mechanistically, the initial and intermediate stages of these kinetics are expected to be controlled by CTB diffusion in solution.

The relationship between CTB coverage and vesicle size was evaluated by plotting the total increase in scattering ( $\Delta I_{\text{s}}=I_{\text{s},\text{v}+\text{CTB}}-I_{\text{s},\text{v}}$ ) and fluorescence (Δ $I_{\text{f}}=I_{\text{f},\text{CTB}}-0$ ) intensities of individual vesicles after CTB binding versus the initial scattering intensities of the vesicles, $I_{\text{s},\text{v}}$ (Fig. 3(b)). In a logarithmic representation, a principle-component analysis of $\Delta I_{\text{s}}$ and $\Delta I_{\text{f}}$ exhibited linear regression lines with slopes of 1.0 and 0.6, respectively.

Physically, $\Delta I_{\text{f}}$ is proportional to the product of the total number of CTB molecules bound to the vesicle and $\eta_{\text{f}}$ . After CTB binding, this his number is proportional to the square of the vesicle radius, $\propto \;r_{0}^{2}$ , and we consequently have

(14) $$\Delta I_{\text{f}}\propto r_{0}^{2}\eta_{\text{f }}.$$

For vesicles without CTB, by analogy, we may write the scattering signal as

(15) $$I_{\text{s},\text{v}}\propto L_{\text{b}}^{2}r_{0}^{4}\eta_{\text{s }},$$

where and $L_{\text{b}}$ is the thickness of the lipid bilayer leaflet of the vesicle. For vesicles with CTB, the correction factors are nearly the same as those in Eq. (15) because the thickness of the CTB layer is much smaller than $r_{0}$ , and accordingly we may write

(16) $$I_{\text{s},\text{v}+\text{CTB}}\propto {\left(L_{\text{b}}+L_{\text{CTB}}\right)}^{2}r_{0}^{4}\eta_{\text{s }},$$

where and $L_{\text{CTB}}$ is the thickness of the colera toxin layer. Thus, the dependence of $I_{\text{s},\text{v}+\text{CTB}}-\;I_{\text{s},\text{v}}$ on $r_{0}$ is the same as in Eq. (15), i.e.,

(17) $$\Delta I_{\text{s}}\propto r_{0}^{4}\eta_{\text{s }},$$

This simple analysis indicates that in logarithmic coordinates the slope in the dependence of $\Delta I_{\text{s}}$ on $I_{\text{s},\text{v}}$ should be 1 (as is observed for the blue data points in Fig. 3(b)), irrespective of the values of the correction factors for scattering ( $\eta_{\text{s}}$ ). For $\Delta I_{\text{f}}$ on $I_{\text{s},\text{v}}$ , however, the corresponding slope depends to extent on the ratio between the correction factors $\eta_{\text{s}}$ and $\eta_{\text{f}}$ . Assuming $\eta_{\text{s}}\approx \eta_{\text{f}}$ the simple analysis should result in a slope close to 0.5, which is indeed close the measured value of 0.6 (red data points in Fig. 3(b)). A more rigorous analysis that accounts for both $\eta_{\text{s}}$ and $\eta_{\text{f}}$ , however, results in an expected slope of 0.6 (see inset in Fig. S7a in Supplement 1), which further supports the validity of our approach.

Hence, this analysis indicates a good consistency between theory and experimental data and furthermore provides credence for quantifying protein binding to vesicle-like structures in terms of absolute film thickness. To describe bare vesicles, one can in principle use Eqs. (8–11) obtained in the thin-shell limit. In the presence of CTB, however, the increase in shell thickness can be considerable, which in turn has an appreciable impact on the magnitude of $\eta_{\text{RGD}}$ (see Sec. 5 in Supplement 1). For this reason, we have used the rigorous spherical core-shell correction factors given in Eqs. (12) and (13) in order to quantify the CTB binding. In accordance with Eq. (4) the scattering intensity from a vesicle of outer radius $r_{\text{v}}$ and lipid leaflet of thickness $L_{\text{b}}$ is given by

(18) $$I_{\text{s},\text{v}}=B\alpha_{\text{v}}^{2}I_{0}(r_{\text{v}},L_{\text{b}})\int_{\phi }\int_{\theta }\sin^{2}\theta \eta_{\text{s}}(r_{\text{v}},r_{\text{v}}-L_{\text{b}})\sin\theta d\phi d\theta ,\;$$

where $\alpha_{\text{v}}$ is the polarizability of a shell-shaped lipid bilayer forming the membrane of the vesicle, with the integration being performed over the acceptance angle of the microscope objective used.

Upon CTB binding, both the thickness and polarizability of the shell region changes and consequently the scattering from a vesicle with bound CTB is given by

(19) $$I_{\text{s},\text{v}+\text{CTB}}=B\alpha_{\text{v}+\text{CTB}}^{2}\;I_{0}(r_{\text{v}},L_{\text{b},}L_{\text{CTB}})\int_{\phi }\int_{\theta }\sin^{2}\theta \eta_{\text{s}}(r_{\text{v}}+L_{\text{CTB}},r_{\text{v}}-L_{\text{b}})\sin\theta d\phi d\theta ,$$

where $L_{\text{CTB}}$ is the thickness of the bound CTB layer and $\alpha_{\text{v}+\text{CTB}}$ is the polarizability of the shell-shaped lipid-CTB layer.

**Fig. 4.**
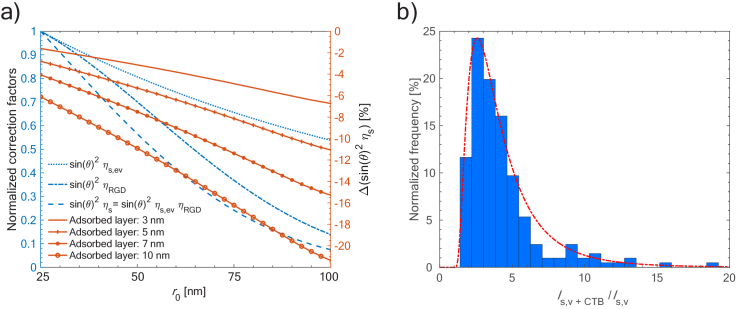
a) Blue: Correction factors for lipid vesicles with the leaflet thickness of 4.5 nm in water, integrated over the acceptance cone of an oil immersion objective with NA 1.0 for a waveguide with a penetration depth of d≈100 nm, excited with a 488 nm TE-polarized laser. Orange: Relative changes in combined correction factors, $\sin^{2}\theta \;\eta_{\text{s}}=\sin^{2}\theta \;\eta_{\text{ev},\text{s}}\eta_{\text{RGD}}$ , after adsorption of a thin adlayer of various thicknesses. b) Histogram of $I_{\text{s},\text{v}+\text{CTB}}/I_{\text{s},\text{v}}$ and a fitted lognormal distribution giving a modal value of 2.6.

The contribution of the correction factors [Eqs. (12) and (13)] to the total scattering intensity is depicted in Fig. 4(a). The blue curves represent the normalized individual ( $\eta_{\text{s}}=\eta_{\text{RGD}}$ or $\eta_{\text{s}}=\eta_{\text{s},\text{ev}}$ ) and combined ( $\eta_{\text{s}}=\eta_{\text{RGD}}\eta_{\text{s},\text{ev}}$ ) correction factors for bare lipid vesicles as a function of radius assuming a lipid shell thickness of $L_{\text{b}}=4.5$ nm and penetration depth of δ=100 nm. As can be seen, the combined effects of extinction and phase-change on scattering intensities can be considerable. For example, the reduction in the scattering intensity for a vesicle with a 50 nm radius compared with a vesicle with a radius of 25 nm is 50%. In contrast, the combined correction factor of a vesicle varies more modestly upon adsorption of a thin adlayer, as is depicted by the orange curves in Fig. 4(a), showing the relative changes in the combined correction factors upon adsorption of thin adlayers of 3-10 nm to vesicles ranging in radii between 25 nm and 100 nm. When a 3 nm thick layer is adsorbed to a vesicle consisting of a 4.5 nm thick lipid bilayer, the relative change in the combined correction factor for vesicles of 25 nm radius and 100 nm radius is between 2% and 6%, respectively. However, this effect increases with increasing adlayer thickness and vesicle radius, and for a 10 nm thick adlayer the corresponding effect ranges from 6% to over 20% for 25 and 100 nm radii vesicles, respectively.

The radii of the vesicles used in our experiments are distributed between 25-50 nm, with a modal value of ∼ 45 nm. A small protein such as CTB (thickness of $L_{\text{CTB}}\approx 3$ nm), will thus induce only a minimal change (∼2.5% ± 0.5%) to the relative overall correction factor. Hence, when calculating the ratio of scattering signal before and after CTB adsorption using Eqs. (14) and (15), the correction factors can in principle be neglected, resulting in a much-simplified expression relating the change in scattering to the polarizability of the particle, given by

(20) $$\frac{I_{\text{s},\text{v}+\text{CTB}}}{I_{\text{s},\text{v}}}≅\;{\left(\frac{\alpha_{\text{v}+\text{CTB}}}{\alpha_{\text{v}}}\right)}^{2}.$$

The polarizability of a shell-shaped particle is given by $\alpha =3V\varepsilon_{0}\varepsilon_{\text{m}}\frac{\;\varepsilon -\varepsilon_{\text{m}}}{\;\varepsilon +2\varepsilon_{\text{m}}}$ , where *V* is the volume of the shell and ε its permittivity. In the case of vesicle, ε is simply the permittivity of the lipid bilayer, $\varepsilon =\varepsilon_{\text{b}}$ . Upon protein binding, however, ε has to be replaced with an effective permittivity $\varepsilon_{\text{eff}}$ , which is a weighted average of the permittivity of the CTB, $\varepsilon_{\text{CTB}}$ , and $\varepsilon_{\text{b}}$ . This average can be deduced using various approximations that all tend to give comparable outcomes. Here we used the Bruggeman approximation [47], given by

(21) $$\overset{2}{\underset{i=1}{\sum }}f_{i}\frac{\varepsilon_{i}-\varepsilon_{\text{eff}}}{\varepsilon_{i}+2\varepsilon_{\text{eff}}}=0,$$

where $f_{i}$ is the volumetric ratio of the constituent material with the permittivity of $\varepsilon_{i}$ .

Figure 4(b) shows the distribution in scattering ratio $\frac{I_{\text{v}+\text{CTB}}}{I_{\text{v}}}$ for roughly 200 individual vesicles after CTB binding had reached saturation. The distribution is fitted with a log-normal function revealing a modal value of 2.6. At saturated coverages, the CTB footprint should cover roughly 50% of the vesicle’s surface area [48] and the total CTB volume thus equates to the combined footprint-area times the CTB thickness. Assuming a lipid bilayer thickness of $L_{\text{b}}=4.5$ nm [49,50], an average vesicle radius of 45 nm, and a refractive index of $n_{\text{b}}=1.48-1.49$ [31] and $n_{\text{CTB}}=1.58-1.60$ [32] for the lipid bilayer and CTB respectively, Eq. (20) and (21) translate the 2.6 scattering ratio into approximately 2.9±0.3 nm CTB thickness, which is in good agreement with the reported thickness of CTB found the literature [41,51]. This close agreement confirms the validity of the proposed approach and demonstrates its potential for unraveling protein adsorption dynamics solely from an increase in scattering intensity. Consequently, in the case of adsorption of a protein with a known thickness and permittivity, the proposed approach can explicitly translate the change in scattering intensity during adsorption to protein coverage.

One should note that Eq. (20) can only be considered a good approximation for small vesicles ( $r_{0}<100$ nm) and thin adlayers (below ∼3 nm), in which case the relative changes in combined correction factor remains negligible within the vesicle size distribution. However, although the relative change in the combined correction factors after adsorption of an adlayer may be significant, it may for a monodisperse vesicle population with a narrow size distribution, be considered almost constant within the vesicle size distribution. In that case the right-hand-side of Eq. (20) can be modified to include a constant corresponding to the ratio of correction factors and be used to estimate the adsorbed layer thickness, further expanding the validity of the simple model to thicker adlayers and its use to estimating the coverage of proteins of known thickness on lipid nanoparticles.

## 5. Conclusion

Transparent waveguide evanescent-field microscopy has here been adapted for use with an inverted microscope and employed for microfluidic assisted liquid control to measure time-resolved fluorescence- and scattering intensities from polystyrene beads and for real time label-free monitoring of protein binding to lipid vesicles.

Approximate analytical models, capable of describing both scattering and fluorescence intensities of polystyrene beads interacting with an evanescent field were proposed and verified by comparing them to measured intensities from surface adsorbed fluorescent polystyrene beads, with radii ranging from approximately 25 nm to 100 nm. The models successfully account for the influence of both extinction and phase changes on the measured intensities from these uniform spherical particles.

Nanoparticles shaped as concentric spheres, with distinct optical properties, such as lipid vesicles, are often more suitably represented using a core-shell model. A simple analytical model describing the scattering of a core-shell particle was constructed. The model was applied to interpret the increase in scattering intensity upon binding of cholera-toxin B sub-unit to ∼ 45 nm in radius POPC vesicles containing 4% G_M1_ immobilized on the waveguide surface. By presenting the data as a ratio between intensity after and before protein binding, the analyzed data could be used to deduce the thickness of the cholera-toxin protein layer with an estimated accuracy of ∼2.5% ± 0.5%.

The general feasibility of this self-consistent means to quantify the thickness of adsorbed protein layers on lipid vesicles, without the need for compensating for uncertainties pertained to possible inhomogeneous illumination profiles, is shown to be better than ∼20% for film thicknesses and vesicle-radius up to ∼10 and ∼75 nm, respectively. Although the analysis was in this work focused on quantifying protein binding in terms of film thickness, revealing sub-nm resolution of the system, the values can be readily converted to bound protein mass [28] as usually desired when the evaluating the stochiometric relation between different binding partners. This suggests that the waveguide chip fabricated on a transparent substrate for compatibility with microfluidic liquid control and inverted microscopes, may pave the way for quantitative fluorescence and label-free scattering microscopy of biological nanoparticles.

## Data Availability

Data underlying the results presented in this paper are not publicly available. These data may be obtained from the authors upon reasonable request.

## Supplemental document

See Supplement 1 for supporting content.
