# Supplementary material for: Label-free quantification of protein binding to lipid vesicles using transparent waveguide evanescent-field scattering microscopy with liquid control
Source: Biomed Opt Express. 2023 Jul 10;14(8):4003–16. doi: 10.1364/BOE.490051 (PMC10549727; doi:10.1364/BOE.490051)
Supplement: Supplementary file 1 [file boe-14-8-4003-s001.pdf]

Supplemental Document

Biomedical Optics EXPRESS

Label-free quantification of protein binding to lipid vesicles using transparent waveguide evanescent-field scattering microscopy with liquid control: supplement

MOKHTAR MAPAR,[1] MATTIAS SJÖBERG,[1,2] VLADIMIR P. ZHDANOV,[1,3] BJÖRN AGNARSSON,[1,2] AND FREDRIK HÖÖK[1,*]

[1] *Division of Biological Physics, Department of Physics, Chalmers University of Technology, SE-41296 Göteborg, Sweden*
[2] *Nanolyze AB, BioVentureHub, Pepparedsleden 1, SE-43183 Göteborg, Sweden*
[3] *Boreskov Institute of Catalysis, Russian Academy of Sciences, Novosibirsk 630090, Russia*
[*] *fredrik.hook@chalmers.se*

This supplement published with Optica Publishing Group on 10 July 2023 by The Authors under the terms of the Creative Commons Attribution 4.0 License in the format provided by the authors and unedited. Further distribution of this work must maintain attribution to the author(s) and the published article's title, journal citation, and DOI.

Supplement DOI: https://doi.org/10.6084/m9.figshare.23560395

Parent Article DOI: https://doi.org/10.1364/BOE.490051

Label-free quantification of protein binding to lipid vesicles using transparent waveguide evanescent-field scattering microscopy with liquid control: supplemental

1. Fabrication of waveguide chip 2
2. Size distribution of fluorescent polystyrene beads 5
3. Fluorescence intensity of polystyrene beads measured under EPI illumination 5
4. Fluorescence and scattering as a function of objective numerical aperture 6
5. Correction factors 8
6. Vesicle dynamics prior to cholera toxin binding evident in scattering 11
7. Amount of collected scattered light 13
8. References 14

1. Fabrication of waveguide chip

The chip fabrication procedure largely follows the methodology described in Refs. [1,2] where one can find a comprehensive characterization of the optical properties and performance of the waveguide, as well as additional information on specific fabrication steps, including atomic force microscopy (AFM) images and profilometry plots showcasing the surface of the core layer and the underlying CYTOP layer.

Prior to fabrication, a 4 inch, 180 μm thick glass substrate (D263 Glass from Valley design Corp.) was cleaned in a mega-sound bath containing base-piranha (1:1 ratio of ammonium hydroxide (15%) to hydrogen peroxide (15%)). After cleaning, the wafer was heated for 20 minutes at 180 °C on a hotplate to rid the surface of residing water molecules. The surface was then functionalized by spin-coating a freshly prepared solution of a 0.2%v APTES (3-Aminopropyltriethoxysilane 99%, Sigma-Aldrich) in 95% ethanol prepared by diluting 99.8% ethanol (Sigma-Aldrich) with deionized water. The spin-coating was followed by 2 minutes of baking at 120 °C on a hotplate (Fig. S1(a)). Type-A CYTOP (CTX-809AP2, AGC Chemicals) was subsequently spin-coated to the required thickness after which the wafer was placed under a glass beaker and baked in a high temperature furnace. After baking the AFM-acquired root-mean squared surface roughness of the CYTOP layer was between 0.5-1.5 nm (depending on scan size).

To achieve a good uniformity, the baking was done in 3 steps, with 2.2 °C/min temperature ramping between steps, in a high temperature furnace under a glass beaker. The substrate was incubated at 50 °C for 1 hour followed by baking at 80 °C for another hour and then for 2 hours at 250 °C before being allowed to cool down to room temperature overnight. The CYTOP surface was then made hydrophilic by depositing 20 nm of aluminum in an electron-beam evaporator (Kurt J. Lesker Co.). Prior to core layer deposition, the aluminum layer was removed in ma-D331 photoresist developer (micro resist technology GmbH) and the substrate subsequently rinsed thoroughly to reveal a hydrophilic CYTOP layer [Fig. S1(b)]. Spin-on-glass (SOG) (IC1-200, Futurrex Inc.) was spun to 480 nm and baked at 120 °C in a conduction vacuum-oven for 24 hours before being allowed to cool down to room temperature [Fig. S1(c)]. The root-mean squared surface roughness of the SOG layer was generally evaluated to values between 0.18 to 1 nm (depending on scan size).

The SOG layer was then functionalized with APTES using the same protocol as before and subsequently a second CYTOP cladding layer was spin-coated using the same parameters as were used for the lower cladding [Fig. S1(d)]. The top cladding was baked in a conduction vacuum-oven at 50 °C for 30 minutes and then at 80 °C for another 30 minutes before a 1-hour baking at 100 °C. The wafer was then left to cool down in vacuum overnight. The top CYTOP cladding was afterwards made hydrophilic using aluminum deposition and removal as before, followed by photolithography defining the microfluidic channels (photoresist, ma-P1225, from micro resist technology GmbH) [Fig. S1(e)]. The photoresist was developed in maD331 [Fig. S1(f)] and the exposed CYTOP etched in a reactive ion etching chamber (Oxford Plasmalab 100) at 50W [Fig. S1(g)] using oxygen plasma. The etching was monitored with laser interferometry and terminated shortly after the core layer of the waveguide was reached [Fig. S1(g)].

When light is being coupled into the waveguide structure by means of but-coupling an optical fiber to the waveguides end-facet, some light may leak into the supporting glass substrate and increase background light intensity. To mitigate this effect, a thin light-absorbing layer was patterned on the back side of the substrate. For this we used a mixture of GMC1060 (Gersteltec), SU-83005 and SU-8 3035 (MicroChem Corp.) that was prepared with similar viscosity to that of GMC1060 and spin-coated to a thickness of 30 μm on the back side of the glass substrate [Fig. S1(h)]. The layer was then soft baked at 65 °C and 90 °C for 10 and 25 minutes, respectively, and exposed for 15 minutes to pattern an opening for easy access of a microscopy objective. The post bake was carried out using the same parameters as for the soft bake. The light-adsorbing SU-8 layer was developed in mr-Dev600

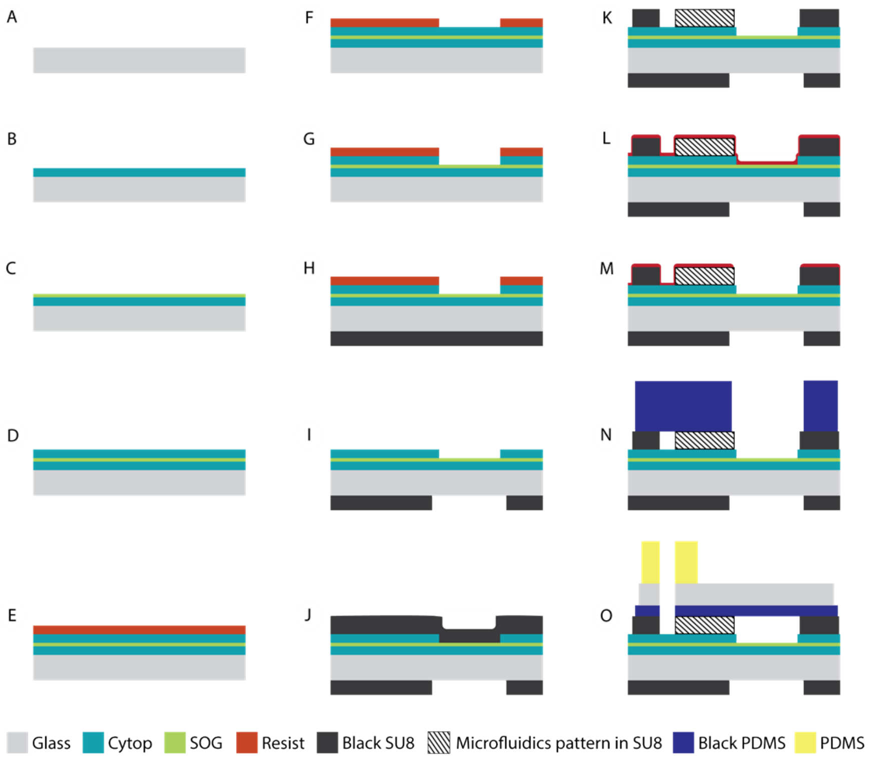

Figure S1. Processing flow of a waveguide device on glass substrate with microfluidics incorporated. a) Substrate preparation for CYTOP coating. b) CYTOP spin-coating and baking. c) Spin-on-glass coating and baking. d) CYTOP spin-coating and baking. e) Spin-coating and exposing the photoresist with the microfluidics and sample-well pattern. f) Development of the photoresist. g) Reactive-ion etching of the upper cladding to form the microfluidic channels in CYTOP. h) Photolithography of black SU-8 on the back side of the substrate defining a window for carrying out microscopy. i) Development of black SU-8 layer and removal of photoresist on top of the substrate. j) Photolithography of black SU-8 on top of the waveguide, with microfluidic pattern slightly wider than the ones in the CYTOP layer. k) Development of the SU-8 layer. At this point the wafer is Spin-coated with a thick photoresist protective layer and diced. l) Spin-coating of a fresh photoresist protective layer to shield the SU-8. m) Patterning the photoresist and oxygen plasma activation of the microfluidic channels. n) Removal of protective layer and placing of a punched-through slab of black PDMS on top of the measurement area. o) If microfluidics is to be used, step n) is skipped and instead of the PDMS slab, a stack of black-PDMS/glass/PDMS with holes for inlet and outlet is thermally bonded to the SU-8 layer.

(micro resist technology GmbH) for 4 minutes, which also resulted in the removal of the positive photoresist on the waveguide-side of the wafer [Fig. S1(i)]. Next, a second layer of light-adsorbing SU-8 was spin-coated on top of the waveguide [Fig. S1(j)] and patterned with an identical but slightly wider pattern compared with that previously formed on the cladding [Fig. S1(k)]. This layer was devised not only to fine tune the height of the channel, but also to compensate for the possible tension buildup by the SU-8 layer on the back side and to additionally provide a possibility to form an adhesive bonding as a sealing strategy for the subsequently formed microfluidic channels.

The waveguide was then protected with a thick layer of positive photoresist and diced with an R07-SD800-BB200-75 blade (Disco) at a feed rate of 0.5mm/sec and spindle speed of

30 Krpm using a Disco DAD3350 dicing machine. To ensure high-efficient in-coupling of light, the quality (flatness and smoothness) of the facets of the diced chips needs to be maximized. Hence, prior to dicing the chips, the R07 blade was dressed on a GC3000PB50 dressing board (Disco) by making 10 cuts at 10mm/sec followed by another 10 cuts at 20mm/sec at spindle speed of 30 Krpm. After dicing, the protective photoresist was replaced with a fresh ma-P1225 photoresist layer and baked at the same temperature as previously [Fig. S1(l)]. The photoresist was subsequently flood exposed from the backside through the black SU-8 layers, which after development, resulted in an exposed SOG core layer in the microfluidic channels, while the photoresist on top of the SU-8 layer remained [Fig. S1(m)].

Prior to an experiment, the exposed SOG layer is activated with a short oxygen plasma treatment, if necessary, and the photoresist protecting the SU-8 is dissolved in mr-Dev600. Depending on final application, the waveguide chips could be designed to have an open-well configuration [Fig. S1(n)], which allows for easy access to the sensing region, or in a flow-cell configuration [Fig. S1(o)], which allows for microfluidic controlled liquid exchange. In the former case, it is enough to place a thin slab of black PDMS with an opening on top of the device to contain a droplet of sample. In flow-cell configuration, a stack of irreversibly bonded black-PDMS/Glass/PDMS with proper inlet and outlet holes is bonded to the SU-8 defining the channel side walls. For that, the black-PDMS is treated for 10 seconds in O_2 plasma (Oxford Plasmalab 100) at 25W and then immersed in a 1-2% aqueous APTES solution for 20 minutes. After a thorough DIW rinse and drying, the black PDMS is bonded to the black SU-8 on a hotplate set at 80 ^{o}C, by keeping them in conformal contact for at least 10 minutes. A schematic showing a ready-to-use transparent waveguide chip, with and without microfluidic channels, is presented in Fig. S2.

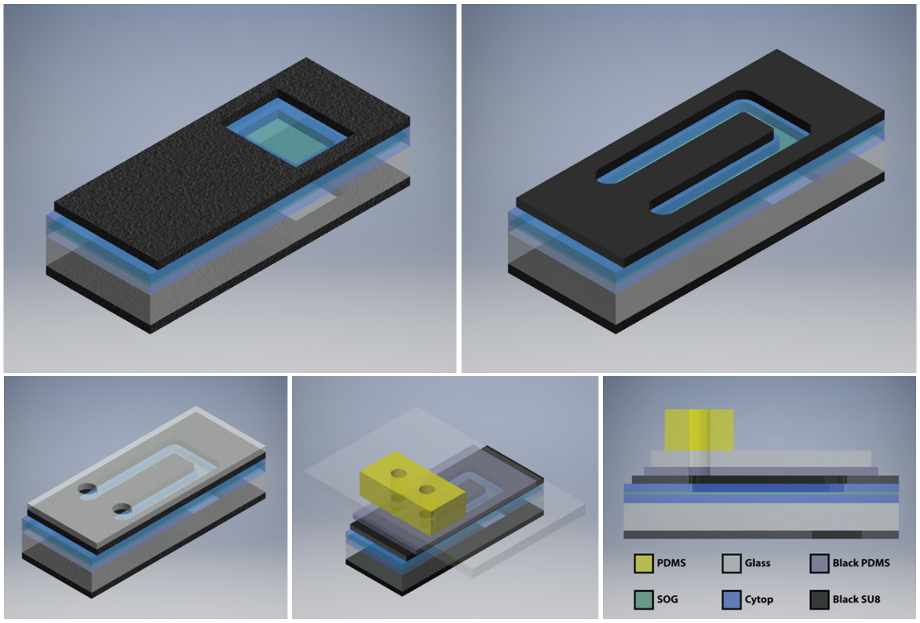

Figure S2 Transparent waveguide chip with two variants of sensing-well configurations intended for open-chamber configuration (top image, left) or closed flow-cell configuration (all images except for top left). The patterned black SU8 on top and bottom of the chip not only helps reducing stray light but also aids with confining the specimen and providing a suitable microscopy window respectively. The microfluidic channel patterned in SU8 was either sealed by bonding to glass directly (bottom left) or to a stack of black PDMS-glass-PDMS (Bottom middle). Bottom right: side view of waveguide with a microfluidic channel after bonding to a stack of black PDMS-glass-PDMS.

2. Size distribution of fluorescent polystyrene beads

Figure S3 shows the measured size distribution for the fluorescent polystyrene beads with nominal diameters 50, 100 and 200 nm as obtained using Nanoparticle Tracking Analysis (NTA) in scattering mode. The beads were found to be normally distributed around 52, 100 and 188 nm, respectively.

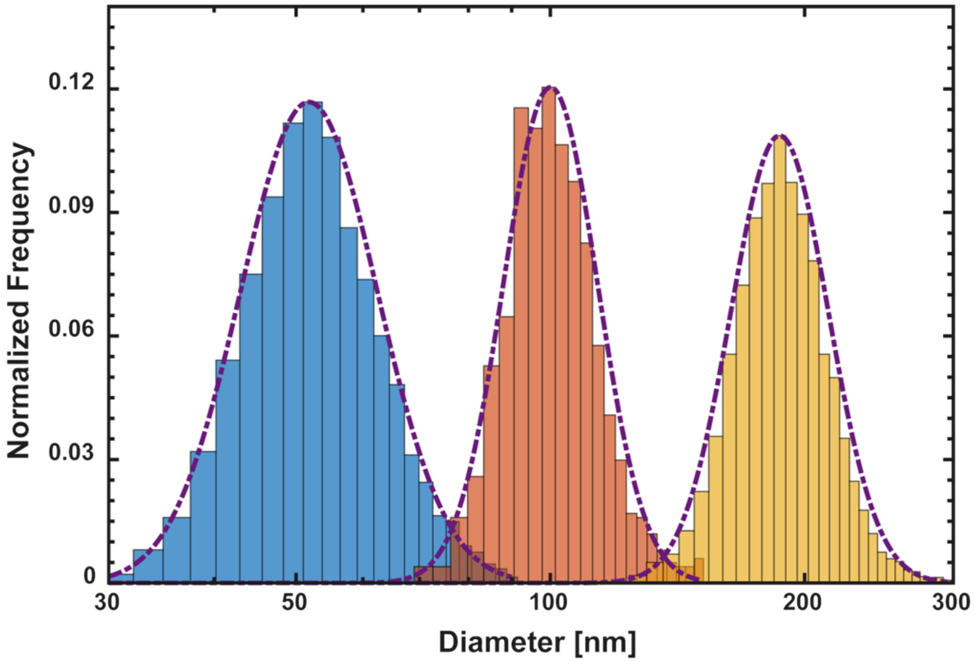

Figure S3 NTA measurement of the size distribution of nominally 50 nm (blue bars), 100 nm (orange bars) and 200 nm (yellow bars) fluorescent beads on a logarithmic diameter axis. The dashed lines show the lognormal distributions for each bead size that on a logarithmic axis appear as normal distributions.

3. Fluorescence intensity of polystyrene beads measured under EPI illumination

The fluorescent beads consist of a mixture of labeled dyes and non-labeled polystyrene material in an unknown ratio. Assuming no self-quenching effects, the fluorescence intensity of the bead is directly proportional to the dye concentration, which corresponds to the total volume of the dyes (V_1) within each bead. On the other hand, the scattering intensity roughly scales with the square of the entire volume of the bead ($V_2{}^2$). While V_2 is known ($V_2 = 4/3\pi r^3$, where r is the particle radius), the volume V_1 is generally unknown. However, it is safe to assume that V_1 is much smaller than V_2 ($V_1 \ll V_2$). Additionally, the quantum yield or fluorescence efficiency is dependent on the particular excitation wavelength used, which in our case (exciting at 488 nm) is suboptimal compared to the ideal excitation wavelength of 542 nm. Together these factors explain the relatively low absolute values obtained for fluorescence intensities compared to their corresponding scattering intensities as observed in Figs 1a and 2 in the main article.

Fluorescent polystyrene beads were adsorbed on the core layer of a waveguide fabricated on a silicon substrate in the same way as done for the experiment presented in the main article and excited using epi-illumination. The beads were divided into three categories corresponding to each nominal bead size based on their measured fluorescence intensity (Fig. S4). Beads

deviating more than 3σ (σ being the standard deviation of the fitted log-normal distribution-function) from the center of the fitted log-normal distribution function were excluded from the analysis. The remaining beads had an average intensity ratio of 33 and 11 for the $\frac{I_{\text{Fluo}}(100\text{nm})}{I_{\text{Fluo}}(52\text{nm})}$ and $\frac{I_{\text{Fluo}}(188\text{nm})}{I_{\text{Fluo}}(100\text{nm})}$ respectively. If the dye concentration was identical for all three bead sizes, one would have expected intensity ratios of 7.5 and 8 respectively, based on the nominal size of the beads. This indicates 4.4 and 6.1 higher average dye concentration in 100 nm and 188 nm beads relative to 52 nm beads, respectively. Hence, to compensate for the uneven dye concentration between the three bead populations, all fluorescence intensity values for the 100 and 188 nm beads presented in the main article were divided by 4.4 and 6.1, respectively.

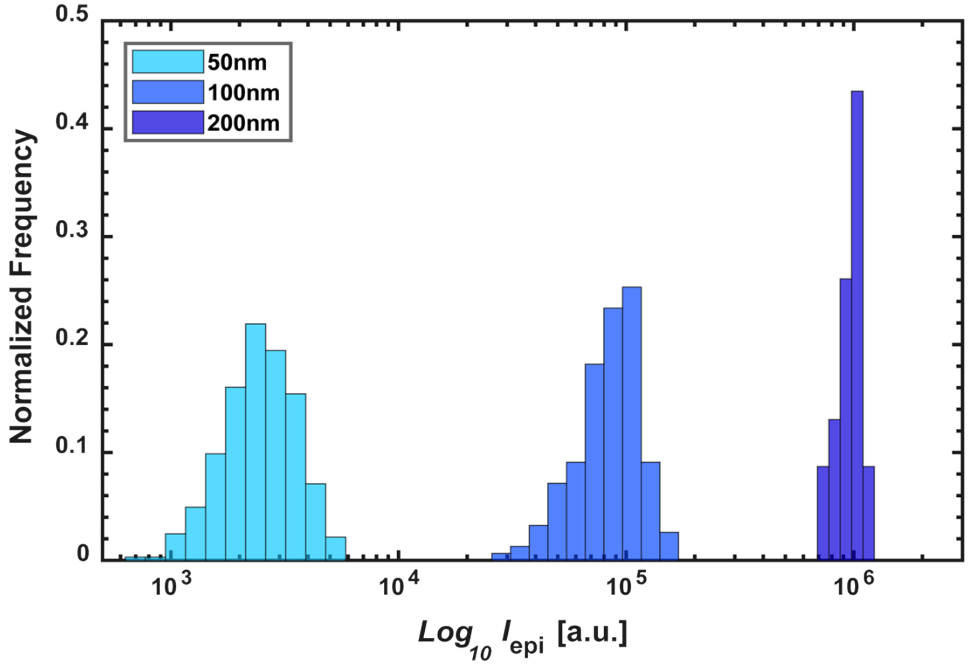

Figure S4 Distribution of the fluorescent intensities of nominally 50 nm (light blue bars), 100 nm (blue bars) and 200 nm (purple bars) polystyrene beads adsorbed on a waveguide core layer under Epi illumination.

4. Fluorescence and scattering as a function of objective numerical aperture

The panels in Fig. S5a display micrographs of fluorescent polystyrene beads with a mean diameter of 188 and 100 nm imaged using 4 and 0.5 second exposure in fluorescence (left panel in Fig. S5a) and scattering modes (right panel in Fig. S5a), respectively, under otherwise identical evanescent illumination and image acquisition conditions. The images share a significant number of objects, although a few are visible only in the scattering image and are attributed to non-fluorescent surface impurities. The influence of the numerical aperture (NA) of the objective on the image quality (Fig. S5b) is evaluated by calculating the signal-to-background ratio (*SBR*) for various NA according to:

$$SBR = \frac{I_{\text{bead}} - I_{\text{back}}}{I_{\text{back}} - I_{\text{dark}}}, \#(S1)$$

where I_{bead}, I_{back} and I_{dark} are the bead (fluorescence or scattering), background and dark-noise intensities, respectively. The highest ratio was obtained for NA 1.0, in scattering and

NA 1.1 in fluorescence while at higher NA the *SBR* decreased as some optical anomalies appeared in the acquired images. However, in some cases, these anomalies that lead to the decrease in SBR, already appeared at slightly lower NA values. Hence, an NA of around 1.0 was used in all our measurements presented in the main article. Figure S5c illustrates the distribution of mean local scattering and fluorescence background intensities per pixel for different sizes of beads, after subtracting the dark noise of approximately 420 counts per pixel. The experimental procedure involved a sequential incubation of beads, starting with 52 nm beads that exhibited the lowest background, followed by 100 nm beads, and concluding with 188 nm beads. The observed elevation in background intensity as the bead size increased can be attributed to two factors: i) the increased total number of neighboring beads acquired during sequential measurements, and ii) the rise in scattering and fluorescence associated with larger bead sizes. This effect is particularly pronounced when considering the largest beads, as they were measured in the presence of other large beads and the pre-existing smaller beads.

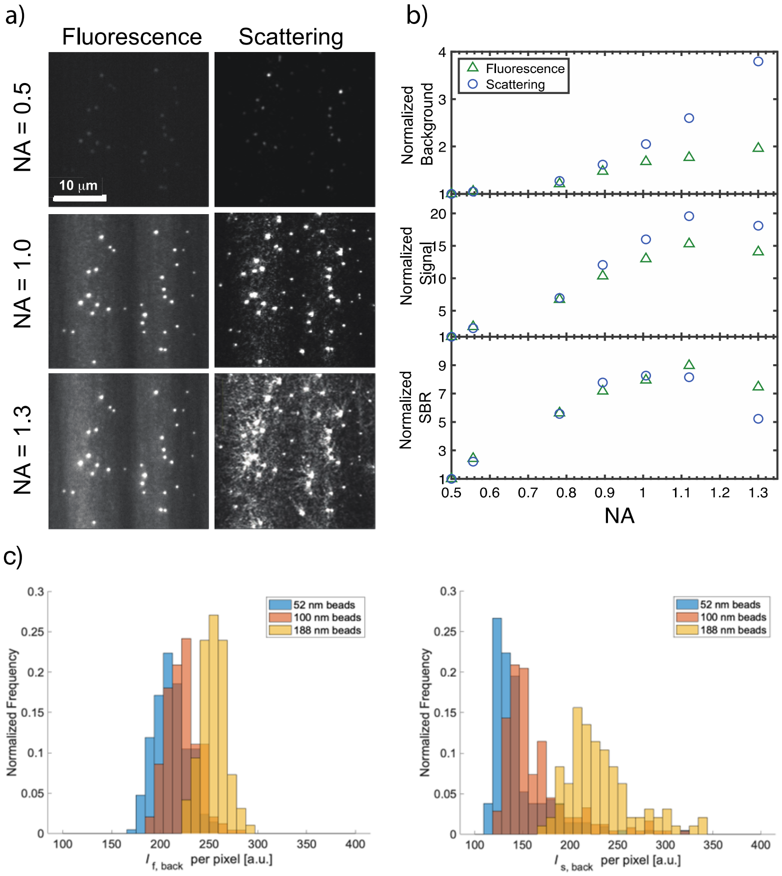

Figure S5 a) 188 and 100nm fluorescent polystyrene beads on the waveguide-chip excited at 488 nm captured using an oil-immersion objective with iris set to different numerical apertures (NA). The images are obtained using 0.5 sec and 4 sec. exposure for scattering and fluorescent, respectively. b) Assessment of waveguide performance based on scattering and fluorescence intensities as a function of NA. The values presented are the average values from over 1000 fluorescent beads. The data, for both fluorescence and scattering, has been normalized to the values obtained at NA =0.5. c) Distributions of local mean background fluorescence and scattering intensities per pixek from polystyrene fluorescent beads of the three different sizes.

5. Correction factors

In our experiments with fluorescent polystyrene beads (radius of 26, 50 and 94 nm) the scattering and fluorescence intensities were simultaneously acquired under evanescent excitation using a 488 nm laser light source. For the evaluation of the intensities, correction factors that accounted for the evanescent excitation were derived [Eqs. (3) and (6) in main article] along with a correction factor based on the form factor of a spherical particle originating from the Rayleigh-Gans-Debye approximation [Eq. (7) in main article] to account for the effect of phase changes for particles with dimensions comparable to the excitation wavelength. It is instructive to have a closer look at these correction factors and briefly analyze their influence on the measured scattering and fluorescence intensities.

In analogy to Fig. 1 in the main article we plot in Fig. S6 the logarithm of the normalized theoretical scattering intensities as a function of the corresponding fluorescence intensities for fluorescent beads with and without the various correction factors assuming a penetration depth of $\delta \sim 100$ nm. When all correction factors are omitted, the curve depicting $\log(r_0/\delta)^6$ and $\log(r_0/\delta)^3$ is linear with a slope of 2, corresponding to the particles behaving as Rayleigh scatterers in homogenous illumination (dotted black curve in Fig. S6a). When the effect of the evanescent field is taken into account [$\eta_{\mathrm{s,ev}}(r_0/\delta)^6$ and $\eta_{\mathrm{f,ev}}(r_0/\delta)^3$], a trend is observed that is only marginally different from that of the dotted curve for small beads but then diverges with increasing bead size (black solid curve in Fig. S6a). For small beads ($r_0 \ll \lambda$) the effect of the evanescent correction factor is expected to be small since the beads experience close to homogenous excitation field over their entire volume. More importantly, since both scattering and fluorescence intensities are affected by an evanescent correction factor, although a bit differently (see Fig. S6b), the net effect is partially cancelled out when plotted against each other.

The effect of phase ($\eta_{\mathrm{RGD}}(r_0/\delta)^6$) is similarly not very noticeable for small bead sizes, but becomes more prominent with increasing bead sizes, which is indeed to be expected in the RGD approximation (green solid curve in Fig. S6a) [3]. Similarly, the combined effect of the two correction factors ($\eta_{\mathrm{s,ev}}\eta_{\mathrm{RGD}}(r_0/\delta)^6$), increases with increasing bead size but shows only minimal effect for beads with radius $r_0 < 60$ nm (red solid curve in Fig. S6a). To better understand how the combined correction factors will effect a linear regression line fitted to the logarithm of scattering and fluorescence data from the three bead sizes used in our experiments ($r_0 = 26, 50$ and 94 nm), we calculate and plot the derivative of $\log((r_0/\delta)^6\eta)$ with respect to $\log((r_0/\delta)^3\eta_{\mathrm{f,ev}})$ for the different correction factors (see inset Fig.S6a). For the three bead sizes the theoretical model predicts a linear regression line with slopes approximately 2.0, 1.9 and 1.5, respectively. Except for the predicted slope of 1.5 for the largest bead size used ($r_0 = 94$ nm), the model is in good agreement with what was obtained in our measurements (Fig. 1 in main text). In fact, the discrepancy at the largest bead size is what one could expect, because in this case the factorization of the correction factors [Eq. (5) in the main text] is not accurate.

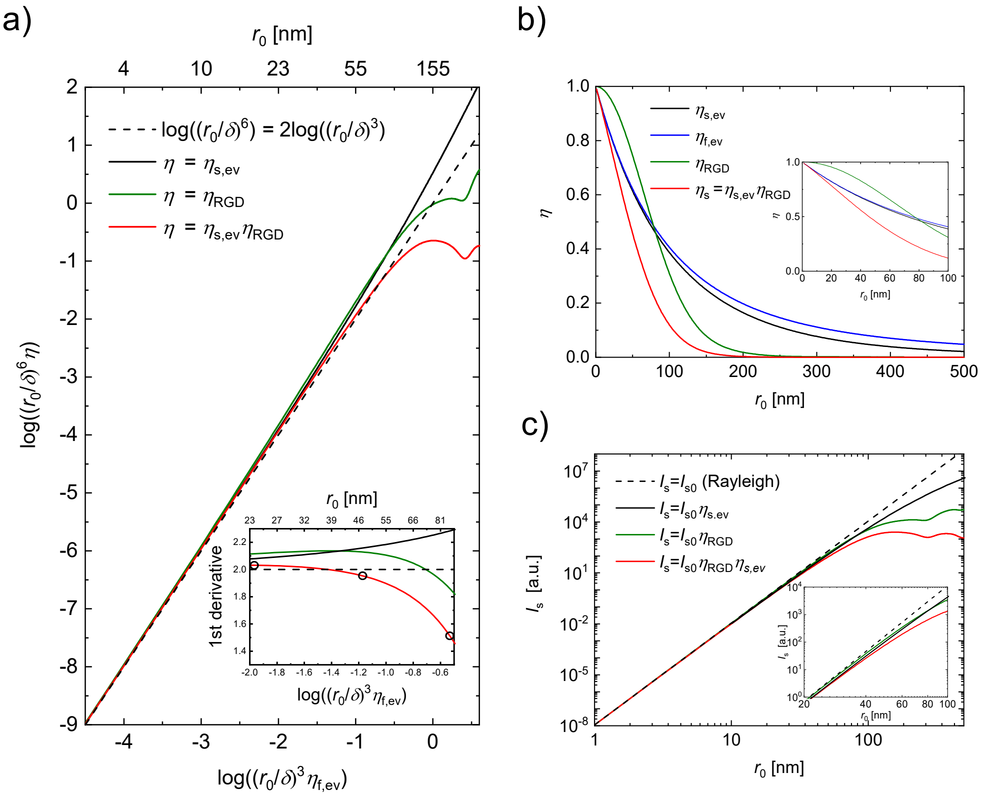

Figure S6 a) Scattering intensity (Eq. 4 in the main article) vs. fluorescence intensity (Eq. 2 in the main article) for fluorescent polystyrene beads (n=1.59) in a logarithmic representation, plotted using the dimensionless factor r_0/δ. The effect of different correction factors ($\eta_{s,ev}$, η_{RGD} and $\eta_{s,ev}\eta_{RGD}$) on the scattering intensity is compared to the r_0^6 vs. r_0^3 dependence expected for a Rayleigh scatterer under homogenous excitation at $\lambda = 488$ nm (black dotted curve). The η_{RGD} correction factor is calculated by integrating Eq. (7) over the collection angle of a 1.0 NA microscope objective and $\eta_{f,ev}$ and $\eta_{s,ev}$, are calculated via Eqs. (3) and (6), respectively assuming $\delta = 100$ nm. The span of the horizontal axis corresponds roughly to $r_0 = 3$ nm to $r_0 = 370$ nm. The inset shows the 1st derivative of the functions with respect to $(r_0/\delta)^3\eta_{f,ev}$ indicating the expected slope of a linear regression line fitted through the datapoints in Fig. 1 in the main article. The hollow black circles indicate the expected slope for $r_0 = 26$ nm, 50 nm and 94 nm, respectively. b) The correction factors as function of bead radius, indicating the similarities between $\eta_{s,ev}$ and $\eta_{f,ev}$ for r_0 up to ~ 100 nm. The inset shows a zoom-in for beads with radii up to 100 nm. c) Effect of different correction factors on the scattering intensity as a function of bead radii. The inset shows a zoom-in for beads with radii between 20 and 100 nm.

Fig. S6b shows the effect of the different correction factors as a function of bead radius using $\delta = 100$ nm. The correction factors for the evanescent excitation on scattering ($\eta_{s,ev}$) and fluorescence ($\eta_{f,ev}$) are indeed very similar for beads with $r_0 < 100$ nm. The RGD correction factor (η_{RGD}) has little impact on beads with small radii, since they tend to scatter in accordance with Rayleigh scattering theory. As the radius increases the impact of the RGD factor increases, and at $r_0 \sim 80$ nm its contribution surpasses the contribution of evanescent correction factor (the η_{RGD} factor is calculated according to Eqs. (8) and (9) in the main article, using $\beta = 0.018$ nm^{-1} and integrating ϑ from $\vartheta = 41°$ to $\vartheta = 139°$).

Fig. S6c shows the effect of different correction factors on the scattering signal of a polystyrene bead as a function of bead radius. Compared to a Rayleigh scatterer with r_0

~ 100 in a homogenous excitation field, both the of evanescent and RGD correction-factors result in a factor ~ 3 reduction in intensity, with the combined effect being almost 10-fold.

In the experiments where Cholera-toxin B subunit was bound to shell-like vesicle-structures we used vesicles with $r_0 \sim 45$ nm and lipid bilayer thickness, $L_\mathrm{b} \sim 4.5$ nm and $n_\mathrm{b} = 1.45$. To interpret this case, we introduced in the main article two sets of correction factors due to phase shift and extinction for fluorescent shell-like particles. One set consisted of simple expression where the finite thickness of the shell-layer was neglected [Eqs. (8)-(10)] while the other set of expressions included the finite thickness of the shell-layer [Eqs. (11)-(13)]. It is interesting to note that the effect of the finite shell-thickness has negligible effect on the extinction correction factors while the opposite holds true for the phase correction (RGD factors). This holds true even for core-shell structures with large shell thicknesses. For this reason, the extinction correction factors given in Eqs. (11) and (13) can in most cases be exchanged for the much simpler expression given with Eqs. (8) and (9). The phase correction factors [Eqs. (10) and (12)], are however more sensitive to both core-shell radius and shell-thickness.

In analogy to Fig. 3b in the main article and Fig. S6 we plot in Fig. S7 the logarithm of the normalized theoretical fluorescence intensities as a function of the corresponding theoretical scattering intensities for fluorescent vesicles with shell thickness $L_\mathrm{b} \sim 4.5$ nm and $n_\mathrm{b} = 1.45$, including and excluding the various correction factors and assuming a penetration depth of $\delta \sim 100$ nm. Compared to Fig. S6, we have now switched the axes to facilitate comparison with Fig. 3b in the main article. If the effect of correction factors is neglected, the fluorescent vs. scattering intensity is represented by a linear curve depicting $\log(r_0/\delta)^4$ and $\log(r_0/\delta)^2$ with a slope of 0.5, corresponding to the vesicles behaving as Rayleigh scatterers in homogenous plane-wave illumination (dotted black curve in Fig. S7a). When the effect of the evanescent field is included [$\eta_\mathrm{s,ev}(r_0/\delta)^4$ and $\eta_\mathrm{f,ev}(r_0/\delta)^2$], the trend is only marginally different from that of the dotted curve for small structures but then diverges with increasing vesicle size (black solid curve in Fig. S7a). For small sizes ($r_0 \ll \lambda$) the effect of the evanescent correction factor is, as expected, small since the structures experience close to homogenous excitation field over their entire volume. Since both scattering and fluorescence intensities include an evanescent correction factor, the net effect is partially cancelled out when plotted against each other (see Fig. S7b) in similar fashion as was observed for the spherical particles (Fig. S6). However, the difference between the respective extinction correction factors is somewhat greater for shell-like structures compared to the spherical structure and hence an increased divergence is observed for shell-like structures compared to spherical structures (compare $\eta_\mathrm{s,ev}$ and $\eta_\mathrm{f,ev}$ in Figs. 6b and 7b)

The effect of phase ($\eta_\mathrm{RGD}(r_0/\delta)^4$) is similarly not very noticeable for small sizes of vesicles, but becomes more prominent with increasing sizes (green solid curve in Fig. S7a) [3]. Similarly, the combined effect of the two correction factors ($\eta_\mathrm{s,ev}\eta_\mathrm{RGD}(r_0/\delta)^4$), increases with increasing vesicle size but shows only minimal effect for vesicles with radius $r_0 < 50$ nm (red solid curve in Fig. S7a). In the inset of Fig. S7a we have plotted the derivative of $\log((r_0/\delta)^2\eta_\mathrm{f,ev})$ with respect to $\log((r_0/\delta)^4\eta_\mathrm{s,ev}\eta_\mathrm{RGD})$. For a vesicle size of $r_0 = 45$ nm the model predicts a linear regression line with a slope of 0.6, which is in good agreement with what was obtained in our measurements (see Fig. 3b in main text).

In Fig. S7b the correction factors are plotted as a function of vesicle outer radius using $\delta = 100$ nm, $L_\mathrm{b} \sim 4.5$ nm and $n_\mathrm{b} = 1.45$. The correction factors for the evanescent excitation on scattering ($\eta_\mathrm{s,ev}$) and fluorescence ($\eta_\mathrm{f,ev}$) are very similar for vesicles with $r_0 < 100$ nm. For vesicles with $r_0 < 100$ nm the extinction correction factor ($\eta_\mathrm{s,ev}$) dominates over the RGD factor (η_RGD) while the opposite is true for $r_0 > 100$ nm.

Fig. S7c shows the effect of different correction factors on the scattering signal of a shell-like lipid vesicle as a function of outer radii. Compared to a Rayleigh scatterer with $r_0 \sim 100$

in a homogenous excitation field, the evanescent and RGD correction-factors result in a factor ~2 and ~7 reduction in intensity, respectively, with the combined effect being approximately 15-fold.

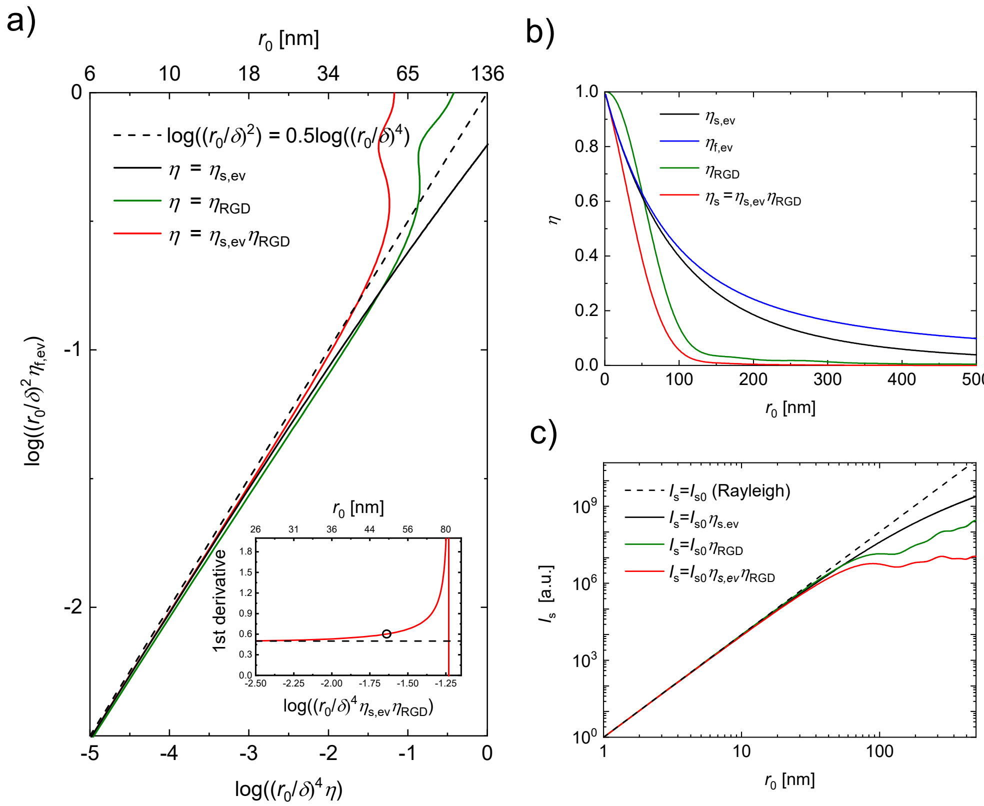

Figure S7 a) Fluorescence intensity vs. scattering intensity for fluorescently labelled lipid vesicles in a logarithmic representation, plotted using the dimensionless factor r_0/δ. The effect of different correction factors ($\eta_{s,ev}$, η_{RGD} and $\eta_{s,ev}\eta_{RGD}$) on the fluorescence intensity is compared to the r_0^4 vs. r_0^2 dependence expected for a Rayleigh scatterer under homogenous excitation at $\lambda = 488$ nm (black dotted curve). The η_{RGD} correction factor is calculated by integrating Eq. (12) over the collection angle of a 1.0 NA microscope objective and $\eta_{f,ev}$ and $\eta_{s,ev}$, are calculated via Eqs. (11) and (13), respectively, assuming $\delta = 100$ nm. The span of the horizontal axis corresponds to $r_0 = 6$ nm to $r_0 = 136$ nm. The inset shows the 1st derivative of $(r_0/\delta)^2\eta_{f,ev}$ with respect to $(r_0/\delta)^4\eta_{s,ev}\eta_{RGD}$, indicating the expected slope of a linear regression line fitted through the datapoints in Fig. 3b in the main article. b) The correction factors as function of bead radius, indicating the similarities between $\eta_{s,ev}$ and $\eta_{f,ev}$ for r_0 up to ~100 nm. c) Effect of different correction factors on the scattering intensity as a function of vesicle radii.

The effect of the correction factors for solid spheres (Fig. S6) and shell-like particles (Fig. S7) can be further visualized by plotting the ratios between them. Using the expressions for solid spheres [Eqs. (3), (6) and (7)] and shell-like particles [Eqs. (11)-(13)], we have in Fig. S8a plotted the ratio $\eta_{f,ev}$ and $\eta_{s,ev}$ and in Fig. S8b the ratio $\eta_{f,ev}$ and $\eta_{s,ev}\eta_{RGD}$, indicating the small effect of the extinction correction factors and the larger effect of the phase correction factors.

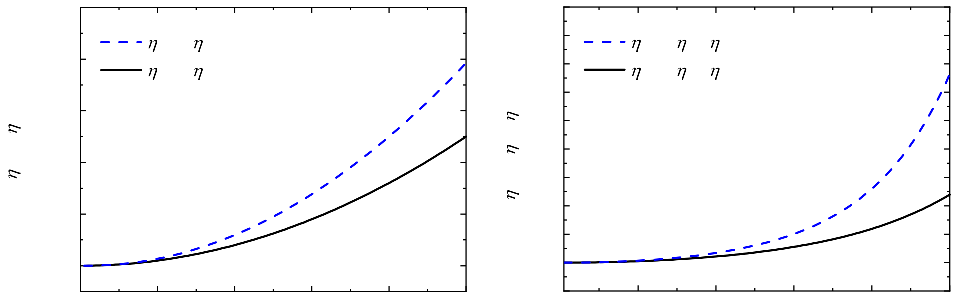

Figure S8 Ratios between fluorescence and scattering correction factors as a function of external radius. a) Fluorescence extinction correction factor ($\eta_{f,ev}$) vs. scattering extinction correction factor ($\eta_{s,ev}$) for 4.5 nm thick shell-like structure [Eqs. (11) and (13)] and a solid spherical structure [Eqs. (3) and (6)]. b) Fluorescence extinction correction factor ($\eta_{f,ev}$) vs. total scattering correction factor ($\eta_{s,ev}\eta_{RGD}$) for 4.5 nm thick shell-like structure [Eqs. (11), (12) and (13)] and a solid spherical structure [Eqs. (3), (6) and (7)]. Here the η_{RGD} correction factors are calculated by integrating Eqs. (7) and (12) over the collection angle of a 1.0 NA microscope objective.

6. Cholera toxin binding evidents in scattering

In the experiments concerning CTB binding to surface-bound lipid vesicles there were evidence of dynamic movements of the vesicles both before and during CTB binding. The vesicles are linked via a NeutrAvidin to a biotinylated polymer surface. The surface had approximately 1×10^8 biotin molecules (acceptors) per cm^2 (mixing ratio of PLL-g-PEG:PLL-g-PEG-biotin = 10.000:1) and are expected to be fixed spatially. The vesicles contained 1 mol% PE-PEG-biotin lipids (in the order of 1000 biotins per vesicle). The bound vesicles may thus be multivalently bound to the surface and should be relatively fixed spatially since the PLL-g-PEG-biotins are immobile on the surface. However, during our measurements, the scattering intensities indicated that the vesicles were to some extent blinking or wiggling, although they did not seem to laterally diffuse in the observation plane (see Fig. S8 for $0 < t < 770$ s). The aforementioned wiggling exhibited greater prominence before the addition of CTB. However, the extent to which the reduction in wiggling can be attributed specifically to the binding of CTB remains a matter of speculation.

The sensitivity of our CTB binding experiments with respect number of CTB molecules per vesicle to can be roughly evaluated by looking at the signal-to-noise ratio (SNR) of our binding curves upon saturated binding. This ratio varies from one vesicle to another but was generally found to be between 4 and 8 (depending on vesicle size and number of available GM1 molecules for binding). The vesicles contain 4 mol% GM1-conjugated lipids, which roughly translates into 3000 GM1 molecules per vesicle, with approximately half of these molecules sitting on the outside of the vesicles (1500 in total). However, not all GM1 molecules are available for binding due to steric hindrance from PEG-biotin molecules on the vesicles and the multivalent binding of PEG-biotins to the surface, limiting CTB access to the vesicle's underside. However, assuming around 1000 available binding sites on a representative vesicle, an SNR ranging from 4 to 8 corresponds to a detection limit of approximately 100-250 molecules. Consequently, the current setup falls short of achieving single-molecule detection limits, although future enhancements in image analysis may improve this outcome.

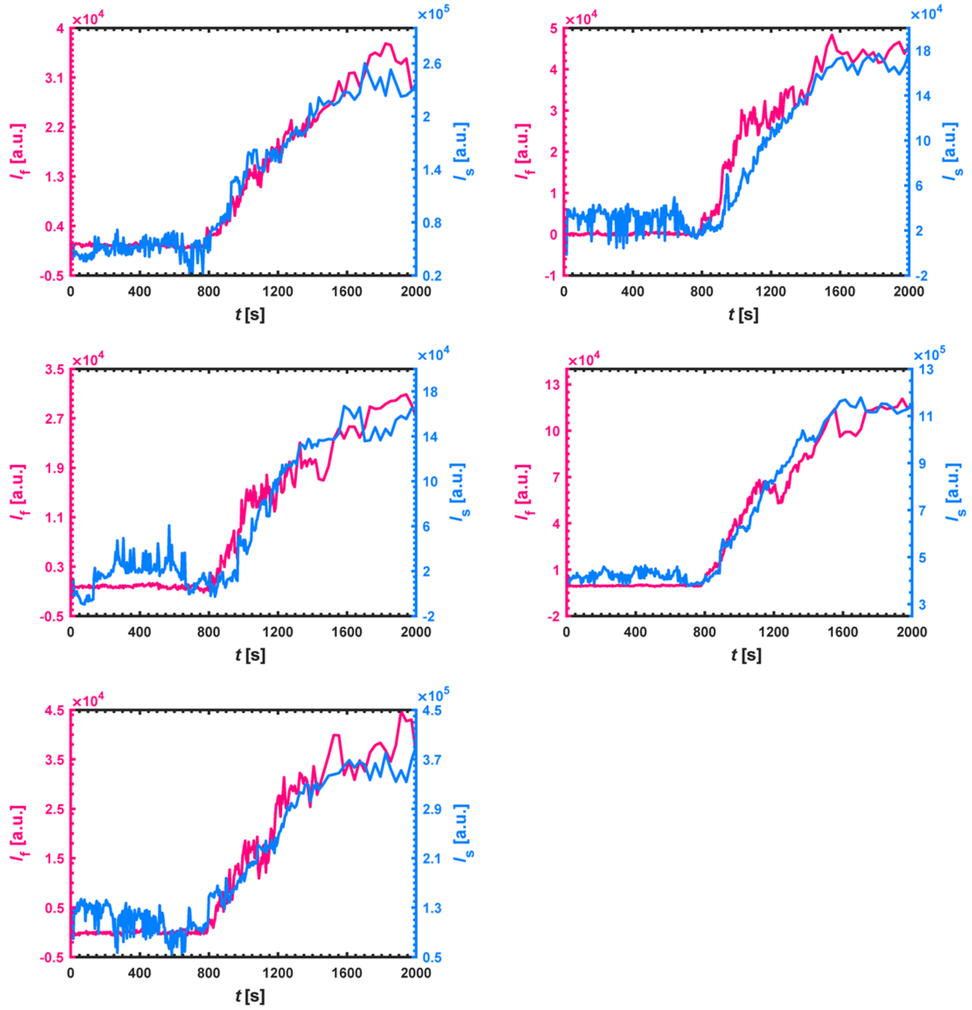

Figure S9 Examples of the CTB binding process for single vesicles in both scatting (blue) and fluorescence (red). The fluctuations in the scattering signal prior to introducing the Cholera toxin (at 770 s) are attributed to vesicle wiggling.

7. Amount of collected scattered light

To estimate the scattered light intensity over the acceptance cone of the objective, a unit solid angles sphere was discretized using a MATLAB code that discretizes the surface of a unit sphere and returns normalized points and weight for Lebedev quadratures, which are in turn used to approximate integrals over solid angles [4]. The incident light was assumed to be along the x-axis (azimuth angle and elevation angle 0°) and the polarization was assumed to be fixed along the y-axis (azimuth angle 90°, and elevation angle 0°). The discrete points within the acceptance cone of the objective represent scattered light vectors collected by the objective. For point/scattered light vectors within the acceptance cone, the scattering plane, i.e. the plane that includes the incident and scattered light, was defined. The polarization vector of the incident light was then split into in-plane and out-of-plane components for each scattering plane. The angle between the scattered light and the out-of-plane polarization component is always 90°, but the angle between the in-plane component and the scattered light changes with the scattered light direction and results in further angular dependency of

the scattered light. This angle is therefore estimated and accounted for when calculating the contribution of the in-plane component on scattering. The resulting contributions of the two perpendicular polarization components in scattering were then added in vectors to obtain the scattered light intensity for each scattering angle, which can be summarized as:

$$I_s(\theta,\varphi) \propto \left(\sin(\pi-\vartheta)\,\eta_s(\theta,\varphi)\sin(\gamma)\right)^2 + \left(\eta_s(\theta,\varphi)\cos(\gamma)\right)^2, \#(S2)$$

where $\vartheta(\theta,\varphi)$ is the angle between the scattered and incident light, $\gamma(\theta,\varphi)$ is the angle between the polarization vector of the incident light and normal vector of the scattering plane, and $\eta_s(\theta,\varphi)$ represents the angular dependence of scattering according to the RGD approximation. Thereafter, the scattering intensity for the scattering angles within the acceptance cone are summed up by taking the Lebedev quadratures weight factors into account.

8. References

1. B. Agnarsson, J. Halldorsson, N. Arnfinnsdottir, S. Ingthorsson, T. Gudjonsson, and K. Leosson, "Fabrication of planar polymer waveguides for evanescent-wave sensing in aqueous environments," Microelectron Eng. **87**(1), 56–61 (2010).
2. B. Agnarsson, M. Mapar, M. Sjöberg, M. Alizadehheidari, F. Höök, F. Hook, and F. Höök, "Low-temperature fabrication and characterization of a symmetric hybrid organic-inorganic slab waveguide for evanescent light microscopy," Nano Futures **2**(2), (2018).
3. H. C. Hulst and H. C. van de Hulst, *Light Scattering by Small Particles* (Courier Corporation, 1981).
4. I. Lebedev and D. N. Laikov, "A quadrature formula for the sphere of the 131st algebraic order of accuracy," Doklady Mathematics **59**(3), 477–481 (1999).
